# Retrospective Study of the Incidence of HFMD and Seroepidemiology of Antibodies against EV71 and CoxA16 in Prenatal Women and Their Infants

**DOI:** 10.1371/journal.pone.0037206

**Published:** 2012-05-25

**Authors:** Feng-Cai Zhu, Zheng-Lun Liang, Fan-Yue Meng, Ying Zeng, Qun-Ying Mao, Kai Chu, Xue-Fang Song, Xin Yao, Jing-Xin Li, Hong Ji, Yi-Ju Zhang, Liang Li, Hong-Xing Pan, Ke Xu, Wei-Ming Dai, Wei-Wei Zhang, Fei Deng, Hua Wang, Jun-Zhi Wang

**Affiliations:** 1 Acute Infectious Disease Control and Prevention, Jiangsu Provincial Center for Disease Control and Prevention, Nanjing, People’s Republic of China; 2 Laboratory 2, National Institutes for Food and Drug Control, Beijing, People’s Republic of China; 3 Shenzhen Kangtai Biological Products Co., Ltd., Shenzhen Science & Industry Park, Guangdong Province, People’s Republic of China; 4 School of Public Health, Southeast University, Nanjing, Jiangsu Province, People’s Republic of China; Johns Hopkins School of Public Health, United States of America

## Abstract

**Background:**

Hand, foot, and mouth disease (HFMD) has been emerging as an important public problem over the past few decades, especially in Asian and Pacific regions. A national program on EV71 vaccine development against HFMD was initiated in China, in 2008, which called for a need for seroepidemiological study for the target population.

**Methodology/Principal Findings:**

This was a retrospective study conducted in Jiangsu Province, in October, 2010. We measured the neutralizing antibodies against EV71 and CoxA16 in a cohort of infants aged of 2, 7, 12, and 27–38 months and their mothers just before delivery. Series sera samples from 975 infants and 555 mothers were collected and analyzed. Questionnaires on the history of HFMD were completed in the survey. A total of 143 HFMD cases were collected, but only 11.2% were reported to the National Infectious Disease Information Management System. The level of maternal antibody titers decreased dramatically during the first 7 month and remained at a relatively low level thereafter. But it increased significantly from month 12 to months 27–38. The accumulate incidence density of HFMD demonstrated a significant increase after 14 months of age, resulting in a accumulate incidence density of 50.8/1000 person-years in survey period. Seropositivity of EV71 antibody in infants at the age of 2 months seems to demonstrate a protective effect against HFMD.

**Conclusions and Significance:**

High seropositive rate of EV71 and CoxA16 antibody was found in prenatal women in mainland China, and there is a need to enhance the HFMD case management and the current surveillance system. We suggest that infants aged between 6 to 14 months should have the first priority to receive EV71 vaccine.

## Introduction

In 1998, a large outbreak of hand, foot, and mouth disease (HFMD) occurred in Taiwan [1]. A total of 129,106 cases were reported, including 78 deaths [2]–[5]. In 2007, more than 80,000 HFMD cases were reported with dozens of deaths in mainland China [6], [7], which arose public concerns around HFMD. Enterovirus 71 (EV71) and coxsackievirus A16 (CoxA16) are two predominant pathogens causing HFMD, though EV71 contributes more to severe and fatal cases [8]–[10].

On May 2, 2008, HFMD was declared a type C legally notifiable communicable disease in mainland China [6], [11]. In the same year, a national program of EV71 vaccine development against HFMD was initiated, which required clinical trials in the target population. Previous studies showed that the HFMD predominantly occurred in children under 5 years old, especially those less than 3 years old. Most adults presented with subclinical infection when exposed to EV71 or CoxA16, and then developed protective antibodies, which can transplacentally pass to newborns [12]–[14]. These transplacental antibodies may protect young infants from infectious EV71 or CoxA16, but they can also impede the effectiveness of certain vaccines and confound interpretation of vaccine-induced immune responses [4], [15]–[17]. For this reason, a better understanding of the dynamic changes in pathogen-specific transplacental antibody and the incidence of HFMD in young infants will be helpful for EV71 vaccine trials with respect to the selection of a suitable target population.

Based on a cohort of healthy neonates enrolled in 2007, we conducted a retrospective epidemiological study on HFMD and the dynamic changes of EV71 and CoxA16 neutralizing antibodies in the infant cohort.

## Materials and Methods

### Subjects and Study Design

In April 2007, a clinical trial titled “The Safety and Immunogenicity of Recombinant Hepatitis B Vaccines in the Healthy Neonates” (ClinicalTrials.gov ID: NCT01183611) was performed. Parturient women and their healthy newborns were recruited in the hospitals of six counties/districts in Jiangsu Province of China. Blood samples were obtained from participating pregnant women before delivery and their infants at 2, 7, and 12 months of age. The first and last baby was born on September 10, 2007 and August 1, 2008, respectively.

In October 2010, we conducted a retrospective epidemiological survey (ClinicalTrials.gov ID: NCT01255124) on the occurrence of HFMD in the infants who had participated in the previous trial mentioned above. Parents or guardians were asked to complete questionnaires on their infants’ health history including the occurrence of blister-like eruptions in the mouth, skin rashes and fever. Associated medical records were also checked. Besides, blood samples were collected from these infants in this survey. Plus the stored sera from the mothers before the delivery and these infants at 2, 7, 12 month of age from the previous trial, all the blood samples were used to measure the EV71 and CoxA16 neutralizing antibody titers. The study was approved by the ethics committee of the Jiangsu Provincial Center for Disease Control and Prevention, and conducted in compliance with the principles of the Declaration of Helsinki. Written informed consent was obtained from the parents or legal guardians.

In this study, HFMD was defined as the occurrence of blister-like eruptions in the mouth or on hand, foot, or buttock, accompanied by fever in the absence of measles, rubella, chicken pox, other febrile eruption disease, or allergy. Non-HFMD infection with EV71 or CoxA16 was defined here as any infant who presented no blister-like eruptions symptoms but were seropositive for EV71 and/or CoxA16 neutralizing antibody after 12 months of age or had at least four-fold increase between the ages of 2 and 7 months. Those infants were deemed as uninfected who presented no HFMD symptoms and had EV71 or CoxA16 neutralizing antibody titers less than 1∶8 at all the 4 time points or the absence of a 4-fold rise between any two consecutive time points.

The purpose of this study was to investigate the infection spectrum of EV71 and CoxA16, the dynamic change of serum antibodies in the infants, and the incidence of HFMD. In order to determine the proportion of underreported HFMD in these six counties and districts, data was also collected from the National Infectious Disease Information Management System which is a passive surveillance system for notifiable communicable disease, from September 2007 through October 2010.

### EV71 and CoxA16 Neutralizing Antibody Assays

For neutralizing antibody detecting in this study, EV71/Fuyang/m01/2008 (C4 genotype) strain was used, which is the predominant genotype in China and also used as the EV71 vaccine strain, while CoxA16/G-10 (A genotype) strain was used to detect the CoxA16 neutralizing antibody. The capability of cross-protection of EV71 and CoxA16 antibody has been studied in previous researches [18], [19].

To measure EV71 and CoxA16 neutralizing antibody by micro-dose cytopathogenic effect, blood samples were initially diluted to 1∶8, inactivated at 56°C for 30 minutes, then serially diluted from 1∶8 to 1∶2048 and mixed with equal volumes of 100 TCID50 EV71 and CoxA16. The mixture was added into a 96-well micro-plate and incubated at 37°C for 2 hours. Finally, RD cell suspension (1×105 cells/mL) was added to the mixture. The plates were then placed in a CO_2_ incubator at 35°C for 7 days, and the cytopathogenic effect was observed under microscopy. Cell control, serum control, virus control, and virus backdrops were all established on each plate. If the backdrops showed 32–320 TCID_50_/well, the test was considered successful. Neutralizing antibody titers were defined as the dilution rate showing 50% inhibition on the cytopathogenic effect. This method had been validated using reference serums of EV71 and CoxA16, and no cross-reaction was found [20]. Neutralizing antibodies equal to or greater than 1∶8 were defined as seropositivity [3], [4], [21], [22].

### Statistical Analysis

The accumulate incidence density of HFMD was calculated by dividing the number of HFMD cases observed by the number of observed person-years of this cohort from birth through October 2010. We used Chi-square testing and Fisher Exact testing to analyze the categorical data, as appropriate. Neutralizing antibody titers below 1∶8 were assumed to be 1∶4 and were log-transformed to calculate the geometric mean titer (GMT) and 95% confidence intervals. Antibody titers >1∶2048 were assigned a value of 1∶2048. Hypothesis testing was conducted using two-sided tests and an alpha value of 0.05 was taken to indicate statistically significant. All statistical analyses were performed using SAS software, version 9.1 (SAS Institute Inc., Cary, NC, U.S.).

## Results

### Subjects

The cohort was first built in April 2007, with 1740 neonates recruited. A total of 975 infants were followed up completely from September 2007 to October 2010, including 548 boys and 427 girls with a sex ratio of 1.3∶1. The average birth weight of the infants was 3502.2 g. Blood samples were obtained from these infants at 2, 7, 12, and 27–38 months of age. There were 555 infants whose mother’s blood samples were also available. The mean age of the mothers was 24.8 years on the date of delivery.

### Infection Spectrum of EV71 and/or CoxA16

**Figure 1:**
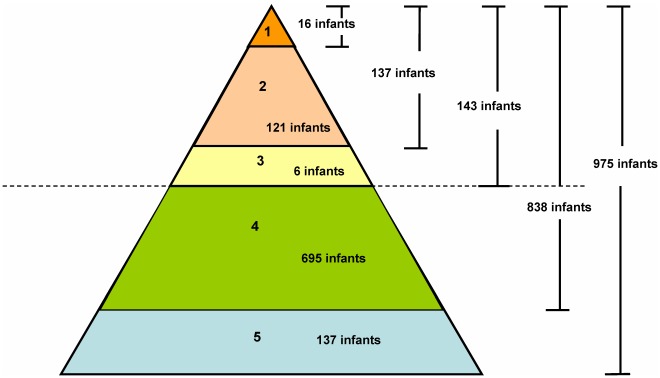
Infection Spectrum of EV71 or CoxA16. 1 Reported cases of HFMD infections from National Infectious Disease Information Management System. 2 Underreported cases of HFMD infections, went to hospital. 3 Underreported cases of HFMD infections, did not go to hospital. 4 Non-HFMD infections of EV71 or CoxA16. 5 Uninfected.

Among the 975 infants, a total of 143 HFMD cases were detected during the retrospective survey period from September 2007 to October 2010 (Figure 1), including 11 inpatients, 126 outpatients, and 6 patients whose families did not seek medical intervention. However, only 16 out of 143 cases were reported to the National Infectious Disease Information Management System with a report rate of 11.2% (16/143). Among the 143 HFMD cases, 99 (69.2%) and 90 (62.9%) cases had EV71 and CoxA16 neutralizing antibody titers increased at least 4-fold after illness, respectively. There were 54 (37.8%) cases had at least 4-fold increase in the two antibody titers, and only 8 (0.6%) cases had neither EV71 nor CoxA16 neutralizing antibody titer increased after illness. Other 695 infants were of non-HFMD infection with EV71 and/or CoxA16, of which 414 (59.6%), 154 (22.1%), and 127 (18.3%) infants demonstrated positive antibody titers for both EV71 and CoxA16, EV71 alone, and CoxA16 alone, respectively. Although up to 85.9% (838/975) of the infants were infected by EV71 and/or CoxA16 during the survey period, only a small number of them showed clinical manifestations of HFMD, and many of the infected infants were asymptomatic. Through this survey period, only 137 (14.1%) infants were not infected with EV71 or CoxA16.

### Dynamic Changes in Seropositive Rate and Accumulate Incidence Density

**Figure 2:**
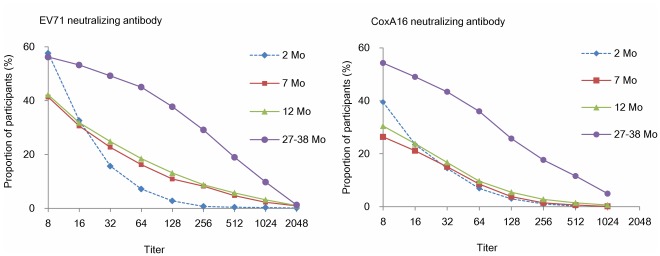
Reverse cumulative distribution curves of neutralizing antibody titers in infants at month 2, 7, 12, and 27–38.

The seropositive rates of neutralizing antibody against EV71 and CoxA16 in prenatal women were 85.3% and 89.1% before delivery, respectively (Table 1). The seropositive rates against EV71 and CoxA16 in the infants at 2 months of age were 57.6% and 39.5%, respectively, which decreased gradually to 41.3% and 26.4% at 7 months of age. Thereafter, the seropositive rates remained relatively low from 7 to 12 months of age. However, a moderate increase in the seropositive rate was observed at 27 to 38 months of age, with 56.2% and 54.3% seropositive rates against EV71 and CoxA16, respectively, and more than 40% of infants had EV71 and/or CoxA16 antibody titers of 1∶32 or more (Figure 2). A similar increase in GMTs was also found from 12 to 27–38 months of age, with EV71 antibody rising to 35.3 and CoxA16 antibody rising to 54.3.

**Table 1:** Dynamic changes of the seropositive rates and GMTs of EV71 and CA16 neutralizing antibodies in mothers and their born children at 2, 7, 12 and 27–38 months of age.

Neutralizing antibody	Mother	Month 2	Month 7	Month 12	Month 27–38
**EV71**	**Seropositive rates % (95%CI)**				
	85.3	57.6	41.3	42.2	56.2
	(82.1–88.1)	(54.5–60.8)	(38.2–44.5)	(39.0–45.3)	(53.0–59.4)
	GMTs (95%CI)				
	25.9	10.2	11.3	12.4	35.3
	(23.4–28.7)	(9.5–10.8)	(10.3–12.5)	(11.1–13.7)	(30.8–40.5)
**CoxA16**	**Seropositive rates % (95%CI)**				
	89.1	39.5	26.4	30.5	54.3
	(86.2–91.5)	(36.4–42.6)	(23.6–29.2)	(27.6–33.5)	(51.1–57.4)
	GMTs (95%CI)				
	31.4	8.1	7.3	8.1	24.3
	(28.4–34.8)	(7.6–8.7)	(6.8–7.8)	(7.5–8.8)	(21.5–27.4)

**Figure 3:**
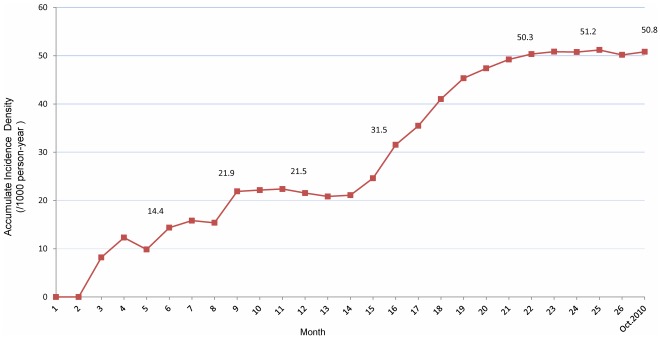
Accumulate Incidence density of HFMD during the observation period in the infant cohort (from the first baby through October 2010).

Among 143 HFMD cases recorded during the epidemiology survey period, the first case was an infant aged 3 months and the last case was an infant aged 34 months. Onset date of HFMD was not available for 2 cases. These data yielded an accumulate incidence density of 50.8/1000 person-years (95% CI: 43.0–59.6) in the survey period (Figure 3). During the first 6 months after birth, only 7 HFMD cases were detected, resulting in an accumulate incidence density of 14.4/1000 person-years. The accumulate incidence density reached 21.9/1000 person-years during the first 9 months and held steadily around 21/1000 person-years until month 14. Thereafter, a significant rise in accumulate incidence density was observed with 31.5/1000, 50.3/1000 and 51.2/1000 person-years at 16, 22 and 25 months of age, respectively.

### Maternal Antibodies and the Occurrence of HFMD in Their Infants

Among 555 infants whose mothers’ blood samples were available, 473 were born to EV71-seropositive mothers and 82 to EV71-seronegative mothers, while 494 infants were born to CoxA16-seropositive mothers and 61 to CoxA16-seronegative mothers. No statistically significant difference in incidence densities was observed between seronegative-mother-born infants and seropositive-mother-born infants. However, serostatus of EV71 antibody in these infants at 2 months of age showed a significant impact on the incidence of HFMD: The EV71-seronegative infants had a higher incidence (P = 0.0228) than EV71-seropositive infants. But no similar result was found regarding serostatus of CoxA16 antibody (Table 2).

**Table 2:** Effects of maternal serologic status on the accumulate incidence density of HFMD in infants.

Antibody		No. ofInfants	Follow-upPerson-year*	No. ofHFMD	Accumulate IncidenceDensity (/1000 person-years)	P Value
**Mothers**	**Anti-EV71**					
	Seronegative	82	237.5	16	67.4 (39.0**–**107.1)	P = 0.1719
	Seropositive	473	1375.0	65	47.3 (36.7**–**59.9)	
	**Anti-CoxA16**					
	Seronegative	61	177.1	8	45.2 (19.7**–**87.1)	P = 0.7286
	Seropositive	494	1435.4	73	50.9 (40.1**–**63.5)	
**Infants at 2 Mo**	**Anti-EV71**					
	Seronegative	413	1193.6	73	61.2 (48.24**–**76.29)	P = 0.0228
	Seropositive	562	1620.4	70	43.2 (33.83**–**54.27)	
	**Anti-CoxA16**					
	Seronegative	590	1697.3	91	53.6 (43.38**–**65.42)	P = 0.4081
	Seropositive	385	1116.7	52	46.6 (34.97**–**60.62)	

* Follow-up person-years were calculated from the first baby through October 2010.

## Discussion

Taiwan has set up HFMD surveillance systems based on both physician and hospital to improve disease management since 1998 [21], [23]–[25]. Mainland China is relatively backward in developing HFMD cases report systems. HFMD was not included in the notifiable communicable disease reporting system until 2008. From this research, we found that HFMD cases reported to the National Infectious Disease Information Management System were far fewer than the actual number of HFMD cases. About 88.8% (127/143) of cases were not reported, highlighting the importance of improving case management and enhancing the sensitivity of current surveillance system. The possible reasons for the high rate of underreporting may include: (1) The symptoms of most cases were mild, and the doctors thought that HFMD was a self-limited infection in infants. (2) The proportion of laboratory-confirmed cases was very low due to unavailable funds and/or facilities. (3) The government and doctors focused more on severe cases, such as pulmonary edema, cardiac insufficiency, and neurologic complications. Therefore, the outbreaks and epidemic status of HFMD we perceived from the current surveillance system was only a small portion of the actual disease burden, like the tip of an iceberg.

In this study, the HFMD cases often lacked pathogenic diagnoses. However, we can make an educated guess regarding the possible prevalence of the pathogenic virus from the observed at least 4-fold increase in the EV71 and CoxA16 neutralizing antibodies before and after illness. The EV71 and CoxA16 were found to be pathogenic virus for most cases of HFMD here.

We found that over 80% of pregnant women had positive serum EV71 neutralizing antibodies, which was higher than the rates in other Asian and Pacific regions [4], suggesting a higher prevalence of EV71 in mainland China. Those infants with seropositive EV71 antibody at month 2 were found to be less likely to contract HFMD. This could be attributed to transplacental maternal antibodies. However, the serologic status of CoxA16 antibody (seropositive or seronegative) did not seem to significantly affect the occurrence of HFMD.

The significant increases in HFMD morbidity and mortality have caused an enormous burden on public health. There are no effective drugs or clinical treatments, and severe cases progress rapidly. Development of a safe and effective vaccine has become the most urgent task to prevent and control this disease. A national program on developing EV71 vaccines has been initiated. Selection of the target population is essential for the vaccine development and the future immunization strategy. Previous study suggested that infants after 6 months of age would be susceptible to both EV71 and CoxA16 infection due to waning maternal antibody, and the vaccines being developed should target infants <6 months of age [4]. Our study showed that the infants aged 7 to 12 months had the lowest seropositivity against EV71 or CoxA16, and the incidence of HFMD remained a low level till month 14, which suggest that infants over 14 months of age would be most susceptible to HFMD. It could be assumed that infants aged 6 to 14 months should have the first priority to receive EV71 vaccine. Considering there are already many doses in routine vaccination within the first 6 months after birth, if we want to apply the EV71 vaccine in the infants under 6 months of age, concomitant administration of EV71 vaccine with current routine pediatric vaccines needs to be assessed in future clinical trial with EV71 vaccine.
